# Lower limb muscle MRI fat fraction is a responsive outcome measure in CMT X1, 1B and 2A

**DOI:** 10.1002/acn3.51979

**Published:** 2024-01-03

**Authors:** Carolynne M. Doherty, Jasper M. Morrow, Riccardo Zuccarino, Paige Howard, Stephen Wastling, Menelaos Pipis, Nick Zafeiropoulos, Katherine J. Stephens, Tiffany Grider, Shawna M. E. Feely, Peggy Nopoulous, Mariola Skorupinska, Evelin Milev, Emma Nicolaisen, Magdalena Dudzeic, Amy McDowell, Nuran Dilek, Francesco Muntoni, Alexander M. Rossor, Sachit Shah, Matilde Laura, Tarek A. Yousry, Daniel Thedens, John Thornton, Michael E. Shy, Mary M. Reilly

**Affiliations:** ^1^ Centre for Neuromuscular Diseases, Department of Neuromuscular Diseases UCL Queen Square Institute of Neurology London UK; ^2^ Roy and Lucille Carver College of Medicine University of Iowa Iowa City Iowa USA; ^3^ Fondazione Serena Onlus, Centro Clinico NeMO Trento Pergine Valsugana Italy; ^4^ Lysholm Department of Radiology National Hospital for Neurology and Neurosurgery London UK; ^5^ Seattle Children's Hospital University of Washington School of Medicine Seattle Washington USA; ^6^ Great Ormond Street Hospital London UK; ^7^ University of Rochester School of Medicine and Dentistry Rochester New York USA

## Abstract

**Objective:**

With potential therapies for many forms of Charcot‐Marie‐Tooth disease (CMT), responsive outcome measures are urgently needed for clinical trials. Quantitative lower limb MRI demonstrated progressive calf intramuscular fat accumulation in the commonest form, CMT1A with large responsiveness. In this study, we evaluated the responsiveness and validity in the three other common forms, due to variants in GJB1 (CMTX1), MPZ (CMT1B) and MFN2 (CMT2A).

**Methods:**

22 CMTX1, 21 CMT1B and 21 CMT2A patients and matched controls were assessed at a 1‐year interval. Intramuscular fat fraction (FF) was evaluated using three‐point Dixon MRI at thigh and calf level along with clinical measures including CMT examination score, clinical strength assessment, CMT‐HI and plasma neurofilament light chain.

**Results:**

All patient groups had elevated muscle fat fraction at thigh and calf levels, with highest thigh FF and atrophy in CMT2A. There was moderate correlation between calf muscle FF and clinical measures (CMTESv2 rho = 0.405; *p* = 0.001, ankle MRC strength rho = −0.481; *p* < 0.001). Significant annualised progression in calf muscle FF was seen in all patient groups (CMTX1 2.0 ± 2.0%, *p* < 0.001, CMT1B 1.6 ± 2.1% *p* = 0.004 and CMT2A 1.6 ± 2.1% *p* = 0.002). Greatest increase was seen in patients with 10–70% FF at baseline (calf 2.7 ± 2.3%, *p* < 0.0001 and thigh 1.7 ± 2.1%, *p* = 0.01).

**Interpretation:**

Our results confirm that calf muscle FF is highly responsive over 12 months in three additional common forms of CMT which together with CMT1A account for 90% of genetically confirmed cases. Calf muscle MRI FF should be a valuable outcome measure in upcoming CMT clinical trials.

## Introduction

Charcot‐Marie‐Tooth disease (CMT) is a clinically and genetically heterogeneous group of conditions characterised by slowly progressive distal wasting, weakness and sensory loss which is usually length dependent. CMT1A due to a 1.4 mB duplication of a region of chromosome 17p11.2 containing the PMP22^1^, ^2^ gene is the commonest subtype accounting for over 50% of all CMT patients in the United Kingdom, Europe and the United States.^3^, ^4^ CMTX1, CMT1B and CMT2A are the next most common forms of CMT. CMTX1 is caused by variants in the gap junction beta 1 (GJB1) gene which encodes the protein connexin 32, which has a role in the formation of gap junction channels in peripheral myelin. Males are more severely affected than females with a usual disease onset between 5 and 20 years of life.^5^ Due to X‐inactivation, manifesting females have much greater variability and typically present in adulthood.^6^ Variants in myelin protein zero (MPZ), which has a role in myelin compaction, cause both demyelinating/hypomyelinating CMT1B and axonal and intermediate subtypes CMT2I and CMT2J.^7^, ^8^ CMT2A is caused by variants in the GTPase protein mitofusin 2, encoded by MFN2. The protein has a role in regulating mitochondrial fusion, transport, mitophagy, mtDNA stability and mitochondria–ER interactions.^9^, ^10^ CMTX1 due to mutations in GJB1 represents 4.8%–12% of all CMT, CMT due to MPZ mutations (CMT1B, CMT2I and CMT2J), is approximately 1.1%–8% of all CMT and CMT2A due to MFN2 mutations is around 3%–7% of all CMT.^4^, ^11^, ^12^, ^13^ Although there are no current treatments for CMT, multiple therapies are in late‐stage development.^14^, ^15^, ^16^, ^17^, ^18^

Over the last decade as therapies are being developed for CMT, a major need has arisen to develop responsive outcome measures (disease biomarkers used to assess the effect of an intervention in clinical trials). Responsiveness is the ability of a measure to quantify change in a prespecified time‐frame.^19^ Developing responsive outcome measures has been challenging in slowly progressive conditions like CMT.^20^ Responsiveness is ideally assessed using an intervention known to be effective; however, there are no such proven treatments in CMT. An alternative is to assess responsiveness by measuring sensitivity to change over time in a natural history, which can be described by the standardised response mean (mean change divided by the change in standard deviation, SRM).^21^ In the original description, large responsiveness is defined as an SRM ≥0.8, moderate responsiveness is 0.5–0.79 and low responsiveness as <0.5.

One of the earliest clinical outcome measures to be developed in CMT is the CMT Neuropathy score (CMTNS), a combined clinical (history and examination) and neurophysiology scale scored 0–36, with a higher score indicating more severe disease. The CMT Examination Score (CMTES) is a sub‐score of the CMTNS, excluding neurophysiology (total 0–28).^22^ Both the CMTNS and CMTES had very low responsiveness over 2 years in the placebo arm of multiple ascorbic acid CMT1A trials.^23^, ^24^ In recent years, multiple other clinical outcome measures (COAs) have been developed (including a modified CMTNS and CMTES (termed CMTNSv2 and CMTESv2))^25^ and a Rasch modified version CMT Neuropathy/Exam Scores (CMTNS‐R/CMTNS‐R)^26^ as well as paediatric and infant scores, the CMT Paediatric Scale (CMTPedS)^27^ and the CMT Infant and Toddler Scale (CMTInfS).^28^ These have been used in natural history studies of CMT1A, CMT1B and CMT2A in adults and, for some forms, in children but at best have found modest progression only a 2‐year period.^29^, ^30^, ^31^

To address the challenge of measuring disease progression over time periods needed for clinical trials, we utilised quantitative lower limb MRI using the three‐point Dixon method to quantify intramuscular fat accumulation and water distribution, two key pathological processes in neuromuscular disorders.^32^ In patients with CMT1A, we previously demonstrated that calf muscle fat fraction increased significantly over 12 months; mean absolute change 1.2% (*p* = 0.002) and we confirmed this finding in a separate CMT1A cohort.^33^ We showed similar responsiveness of intramuscular calf fat fraction in another inherited neuropathy, hereditary sensory neuropathy type 1 (HSN1) which, despite its name, usually has a length dependent motor neuropathy.^34^ While previous publications have looked at qualitative muscle MRI in other CMT subtypes, for example, showing a different distribution of involvement between CMT1A and CMT2A,^35^ to date quantitative studies have been limited to CMT1A and HSN1.

The primary aim of this cross‐sectional and longitudinal study was to study the responsiveness of MRI determined fat fraction at calf and thigh level in adults with CMT due to variants in the GJB1, MPZ and MFN2 genes, compared to matched controls. Secondary objectives included assessment of the validity of intramuscular fat accumulation determined by MRI as a biomarker of disease progression in these patients by correlating it with clinical scores and the plasma axonal biomarker neurofilament light chain (NEFL).

## Subjects/Materials and Methods

### Ethical approvals, study design and patient recruitment

Ethical approvals were gained from the institutional review boards and ethics committees of the participating centres. Informed consent was obtained from all participants. Participants were evaluated between 2018 and 2022. Clinical data were collected prospectively at each study visit.

We aimed to recruit 60 participants aged 16–60 years (at least 20 each with CMTX1, CMT1B and CMT2A) were recruited from the existing inherited neuropathy cohorts at the Queen Square Centre for Neuromuscular Diseases and at the University of Iowa Roy and Lucille Carver College of Medicine as well as age‐ and sex‐matched healthy controls without known neuromuscular disease and with normal neurological examination. (Table 1). Sufficient controls were recruited to allow three subgroups of 20 to match each CMT subtype, with some controls providing data for more than one CMT subtype. Inclusion criteria for this two‐site longitudinal observational study were disease causing variants in the relevant genes affecting the patient, or at the US site, the patient must have a compatible phenotype and nerve conduction study and a pathogenic variant in a first degree relative. Pathogenicity was assessed in a multidisciplinary setting and decision for inclusion was made by the Principal Investigator at each site. Only males were included with CMTX1 due to the greater phenotype variability seen in females resulting from random x‐inactivation. For the purposes of this study all CMT patients with variants in MPZ regardless of the phenotype were termed CMT1B.

**Table 1:** Demographic data in patient and matched control groups and baseline clinical data in patients.

Parameter	CMTX1	CMT1B	CMT2A
Patients: Male:Female	22:0	13:8	8:13
Matched controls: Male:Female	20:0	13:8	8:10
Patients age	40.0 ± 10.9 (24–60)	46.5 ± 12 (18–60)	36.9 ± 16 (16–60)
Matched controls age	38.8 ± 13.3 (18–60)	42.0 ± 11.9 (18–59)	36.9 ± 15.6 (18–59)
CMTESv2 (0–28)	10.8 ± 4 (3–19)	13.5 ± 5.3 (5–24)	12.3 ± 4 (2–19)
CMTESv2‐R (0–35)	14.6 ± 5 (5–25)	17.5 ± 6.9 (6–30)	16.3 ± 4.7 (3–25)
MRC (0–70)	60.9 ± 5.1 (52–68)	60.9 ± 6.9 (49–70)	57.5 ± 12.2 (28–70)
CMT‐HI (0–100)	26.2 ± 19.6 (0.2–69.1)	36.4 ± 24.7 (0–77.4)	40.9 ± 17.1 (15.7–85.9)
ONLS (0–12)	3.2 ± 1.4 (0–5)	3.7 ± 1.5 (1–7)	3.5 ± 2.2 (0–10)

Data given as mean ± standard deviation (range).

Exclusion criteria included safety‐related contraindications to MRI, planned foot surgery or pregnancy and or concomitant neuromuscular disease.

Participants completed a clinical assessment including medical and family history, clinical examination, MRC scoring, CMTESv2, ONLS and CMT‐HI, followed by lower limb muscle MRI [45–60 minutes] at each study visit, that is, at baseline and at 12 months follow up. CMTESv2‐R was calculated. In addition to an overall MRC sum score (0–70, shoulder abduction, elbow flexion, wrist extension, forefinger abduction, hip flexion, knee extension, ankle dorsiflexion scored 0–5 bilaterally), an MRC sum score at the knee (sum of right and left knee flexion and extension, 0–20) and MRC sum score at ankle (sum of right and left ankle plantarflexion, dorsiflexion, inversion and eversion, 0–40) were calculated to correlate with thigh and calf fat fractions, respectively. The sum of the lower limb motor symptoms and examination sub‐scores of the CMTESv2 (0–8) was also calculated to correlate with MRI measures.

All blood samples were taken and processed within 1 h. Blood was collected into EDTA‐containing tubes and centrifuged at 20°C at 3500 rpm for 10 min. Plasma was then aliquoted and stored at −80°C. Plasma sample neurofilament light (NfL) concentration was measured using a commercially available NF‐Light kit on a single molecule array (SimoaTM) HD‐1 instrument (Quanterix).

Three‐point Dixon gradient echo data were acquired at thigh and calf level covering both sides with 10 axial slices centred 20 cm above and 13 cm below the right lateral tibial plateau (London: Siemens Prisma, 3 T, TR = 101 ms, TE = 3.45/4.60/5.75 ms, flip angle 10 degrees, NSA = 4, FOV = 410 × 205 mm, slice gap 10 mm, voxel size 0.8 × 0.8 × 10 mm^3^, Iowa: GE Discovery MR750, 3 T, TR = 101 ms, TE = 3.45/4.60/5.75 ms, flip angle 10 degrees, NSA = 1, FOV = 410 × 410 mm, slice gap 10 mm, voxel size = 0.8 × 0.8 × 10 mm^3^). The fat‐fraction map was based on a two‐component model as described by Glover.^36^ Anonymised imaging data were transferred to the UCL site using the established pipeline where they were quality assured, processed, and entered into the blinded imaging repository by the study physicist (SW). To maintain imaging quality, feedback was typically shared within 48 h. For remote site quality control, a participant was scanned at both sites, and pre and post scanner update scans were performed and analysed at both sites.

**Figure 1:**
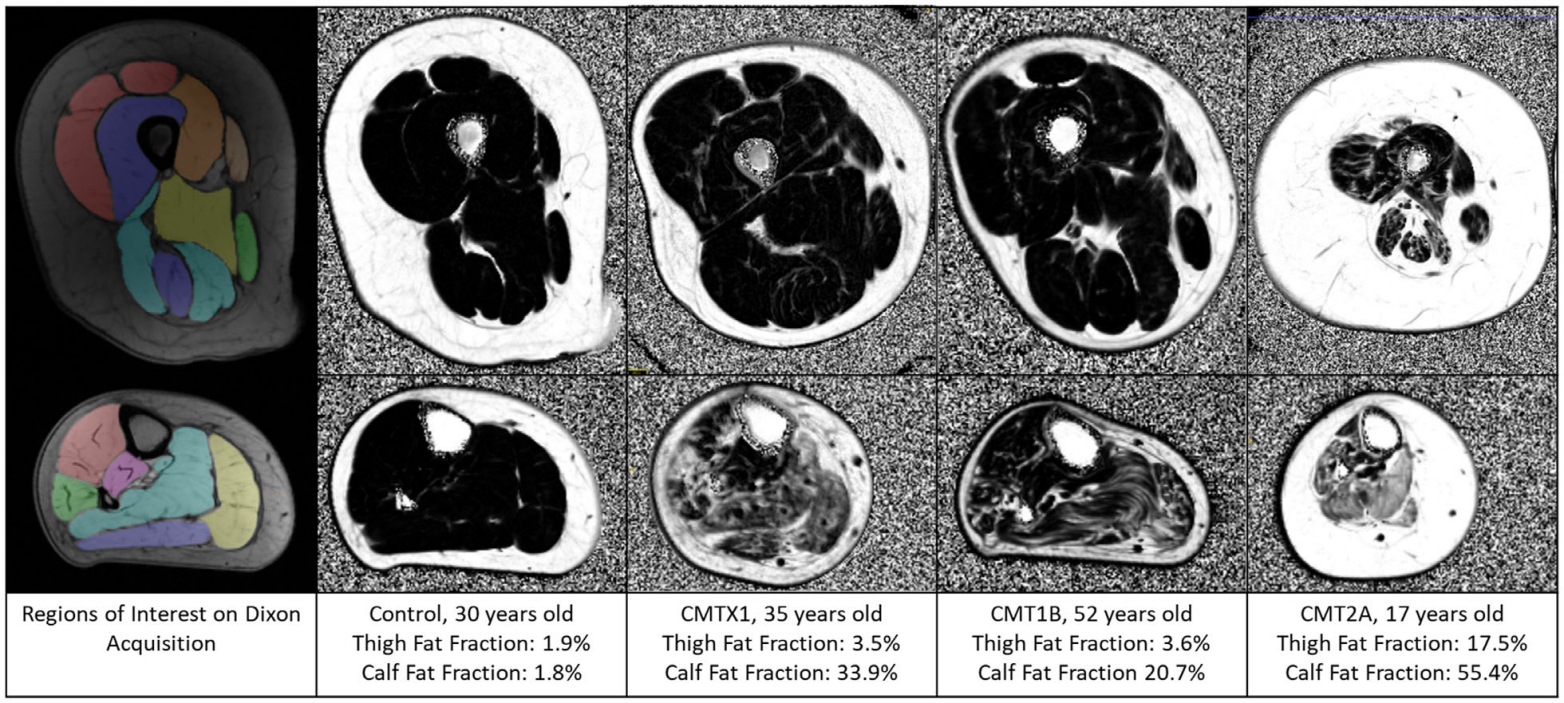
Example axial images of right thigh (top panels) and right calf (bottom panels). The left‐most images are examples of regions of interest drawn on turbo spin echo sequence on which they were drawn (TE = 3.45 ms). Other images are example fat fraction maps from the subject with median calf fat fraction in each group. Fat fraction maps are grey scale black = 0%; white = 100% fat. The control shows low levels of fat fraction at thigh and calf. In CMTX1 and CMT1B patients mild streaking is seen in thigh muscles with a slight increase in fat fraction, with moderate fat accumulation in calf muscles. The CMT2A patient shows fat accumulation and atrophy in thigh and calf muscles.

A trained single observer (CMD) used the open‐source software ITK‐SNAP^37^ to manually label regions of interest (ROI) on a paired mid‐calf and paired mid‐thigh images blinded to visit order and disease versus control participation. A single slice was segmented with the calf slice 13 cm below the tibial plateau, the thigh slice 20 cm above the tibial plateau as identified on a proton density weighted coronal image, with left and right limbs considered separately. At thigh, all muscles except adductor longus were segmented, at calf all muscles were segmented (Fig. 1).

All fat fraction maps were checked for artefacts. Maps with generalised artefact such as movement artefact were not analysed. Isolated voxels with extreme ff values (for example >120%) usually in locations of tendons were manually removed. Data were extracted for individual muscles and for all muscles combined at thigh and calf level using custom written software. Mean fat fraction expressed as a percentage (%ff) and cross‐sectional area (CSA in cm2) were extracted with all muscles in thighs or calves treated as a single segmentation. The contractile cross‐sectional area is derived from the measured fat fraction and total cross‐sectional area: cCSA = CSA × (100 – %ff)/100. Outliers which showed greatest interscan differences were manually checked as to their slice localisation, map quality and segmentation as a final quality control step.

### Statistical analysis

The planned follow up window for all participants was 50–56 weeks; however, the COVID‐19 pandemic began approximately one‐third of the way through the follow up window and resulted in a substantial proportion of unavoidably delayed follow ups. The decision was taken to normalise the data to an interval of 1 year for all participants by dividing the measured change by the interval in years. Change in CSA and cCSA were calculated as percentage change from baseline, annualised as above.

Statistical analysis was undertaken using IBM SPSS 27. Differences in clinical parameters between patient groups were analysed using ANOVA. Data for subgroups versus their respective controls was analysed using Student two‐tailed *t* tests, longitudinal change evaluated with paired *t* tests and Spearman rank correlations were used to assess relationships between MRI and clinical outcome measures. External responsiveness of outcome measures was assessed using the SRM. Subgroup analysis was performed across all patients grouped based on baseline fat fraction at each anatomical level based on our previous study in CMT1A^33^ into normal or near normal fat fraction (<10%), intermediate fat fraction (10–70%) and end‐stage fat fraction (>70%).

## Results

### Participant demographics and clinical findings

A total of 64 patients were enrolled: 22 CMTX1, 21 CMT1B and 21 CMT2A. Thirty healthy controls were enrolled with subgroups of 20 healthy controls selected to be overall age and gender matched to each CMT subtype (Table 1). Baseline clinical data for each patient group showed that overall severity as measured by the CMTESv2 was similar in each patient group (ANOVA *p* > 0.05 for all); however, a wider range of muscle weakness as measured by the MRC score was seen in the CMT2A group (Table 1). Baseline NEFL levels were higher in all patient groups than controls (ANOVA, Tukey) with CMT1B NEFL also significantly higher than CMTX1 patients (*p* = 0.04). NEFL correlated with age in controls and CMT1B patients, but this was not seen in CMTX1 or CMT2A patient groups (Table 4).

### Baseline MRI data

Baseline MRI results for all muscles at thigh and calf levels for each CMT subtype and their matched controls are shown in Table 2. All patient groups showed elevated fat fraction and reduced total and contractile cross‐sectional areas for muscles at calf level compared with their matched control groups. CMT2A had greatest fat fraction (*p* = 0.029 versus other types) and atrophy (*p* < 0.001 versus other types) at calf level with cross‐sectional area half that seen in their matched controls. Thigh muscle fat fraction was also significantly elevated in all patient groups but was greatest in the CMT2A group (*p* = 0.003 versus other types) and the CMT2A group also showed significant reduction in thigh muscle cross‐sectional area compared with their matched controls. Fat fraction maps of the right thigh and calf from a typical subject in each group is shown in Fig. 1.

**Table 2:** Baseline and Longitudinal MRI data in patient groups and matched controls.

Baseline data									
Metric	CMTX1	Controls	*p*	CMT1B	Controls	*p*	CMT2A	Controls	*p*
Thigh FF (%ff)	5.5 ± 5.5 (22)	2.2 ± 1 (20)	0.01	4.5 ± 2.7 (21)	1.9 ± 0.8 (21)	<0.001	19 ± 24.7 (20)	1.9 ± 0.8 (18)	0.006
Thigh CSA (cm^2^)	246.4 ± 48.6 (22)	262.8 ± 49 (20)	0.28	204.3 ± 60.5 (21)	224.3 ± 51.4 (21)	0.26	153.5 ± 51.3 (21)	220.2 ± 55.1 (18)	<0.001
Thigh cCSA (cm^2^)	232.5 ± 46.5 (22)	256.9 ± 46.8 (20)	0.10	194.6 ± 55.5 (21)	220 ± 49.5 (21)	0.06	128.2 ± 61.3 (20)	216 ± 54.1 (18)	<0.001
Calf FF (%ff)	33.4 ± 24.4 (22)	2.3 ± 1.1 (20)	<0.001	26.4 ± 24.8 (19)	1.9 ± 0.8 (21)	<0.001	46.5 ± 20.4 (20)	2.1 ± 1.0 (18)	<0.001
Calf CSA (cm^2^)	89.3 ± 32.3 (22)	143.5 ± 28 (20)	<0.001	100.1 ± 44.9 (20)	123.4 ± 25.5 (21)	0.05	61.0 ± 28.1 (20)	119.3 ± 25.7 (18)	<0.001
Calf cCSA (cm^2^)	61.6 ± 39.7 (22)	140.1 ± 27.1 (20)	<0.001	74.5 ± 43 (19)	120.9 ± 24.5 (21)	<0.001	32.6 ± 20.1 (20)	116.8 ± 25.0 (18)	<0.001

Longitudinal data									
Metric	CMTX1	Controls	*p*	CMT1B	Controls	*p*	CMT2A	Controls	*p*
Thigh FF (%ff)	0.4 ± 1.0 (19)	0.0 ± 0.5 (17)	0.26	0.2 ± 0.8 (20)	−0.1 ± 0.4 (20)	0.35	1.0 ± 1.7 (19)	0.1 ± 0.3 (17)	0.02
Thigh CSA (%)	−2.5 ± 7.1 (19)	−1.0 ± 7.2 (17)	0.1	−3.3 ± 6.7 (20)	−1.6 ± 5.8 (20)	0.05	−5.2 ± 7.8 (20)	−1.2 ± 6.3 (17)	0.005
Thigh cCSA (%)	−2.9 ± 7.6 (19)	−1.0 ± 7.1 (17)	0.09	−3.5 ± 6.6 (20)	−1.5 ± 5.7 (20)	0.03	−5.2 ± 5.2 (19)	−1.3 ± 6.3 (17)	0.005
Calf FF (%ff)	2.0 ± 2.0 (20)	0.0 ± 0.4 (17)	<0.001	1.6 ± 2.1 (18)	0.1 ± 0.4 (20)	0.004	1.6 ± 2.1 (19)	0.1 ± 0.3 (17)	0.002
Calf CSA (%)	−3.7 ± 6.5 (20)	−2.1 ± 6.1 (17)	0.02	−3.1 ± 6.9 (20)	−1.8 ± 5.1 (20)	0.007	−4.6 ± 8.4 (19)	−0.6 ± 7.7 (17)	0.01
Calf cCSA (%)	−6.8 ± 7.9 (20)	−2.1 ± 6.0 (17)	<0.001	−6.6 ± 9 (18)	−1.9 ± 5 (20)	0.001	−7.6 ± 11.3 (19)	−0.7 ± 7.7 (17)	<0.001

Data are presented mean ± standard deviation (n). Longitudinal values are standardised to be change over 12 months. Change in area are expressed as percentage baseline. *p*‐values are Student paired *t*‐tests for patient groups between baseline and follow up. *p*‐Values are Student two tailed *t*‐tests between patient group and matched controls for baseline and paired Student *t*‐test for annual change.

cCSA, contractile cross‐sectional area; CSA, total cross‐sectional area; FF, muscle fat fraction.

**Figure 2:**
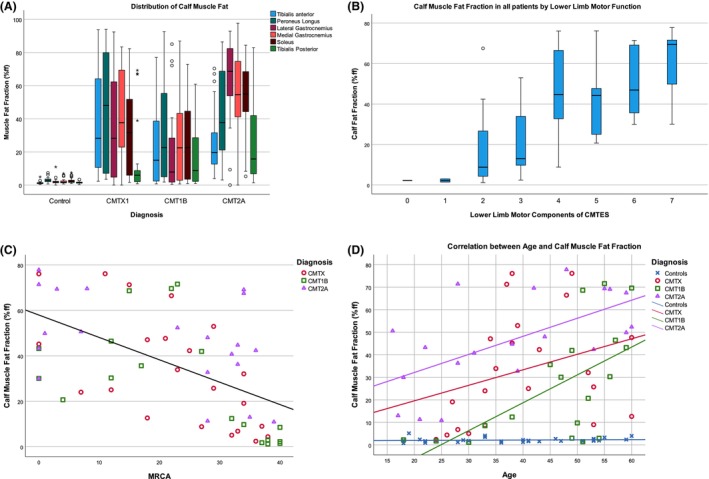
Cross‐sectional MRI‐clinical correlations. (A) Box and whisker plot of muscle fat fraction in right calf muscles by disease group. All 30 control subjects are grouped in this plot. (B) Box and whisker plot of mean calf muscle fat fraction in patients grouped by their lower limb motor components of CMTESv2. Box and whiskers represent median/IQR/range with circled minor outliers and asterisked major outliers. (C) Inverse correlation between calf muscle fat fraction and total ankle MRC grade by disease group (sum ankle plantarflexion, dorsiflexion, inversion, eversion bilaterally and maximum score 40). (D) Correlation between calf muscle fat fraction and age in disease groups and controls.

The distribution of calf muscle fat accumulation varied between groups (Fig. 2A), with CMT2A showing greatest involvement of the muscles in the superficial posterior compartment (soleus and both heads of gastrocnemius), while in CMTX1 peroneus longus was most affected. All patient groups had relative sparing of tibialis posterior. Control subjects had low fat fraction in all muscles as expected.

### Baseline MRI–clinical correlations

All MRI–clinical correlations are reported in Table 3. Considering all patients combined, there were highly significant low to moderate correlations between calf muscle fat fraction and all measures except ONLS MRC knee score, and CMT‐HI which did not correlate. Thigh muscle fat fraction showed similar highly significant correlations to clinical measures except age and ONLS. Correlation between CMTESv2‐R and muscle fat fraction was significant in all patients combined for both thigh (rho = 0.349, *p* = 0.0005) and calf levels (rho = 0.430; *p* = 0.001) and in disease subgroups (CMTX1 calf rho = 0.420, *p* = 0.05; CMT1B thigh rho = 0.529, *p* = 0.014; CMT1B calf rho = 0.729, *p* < 0.001; CMT2A thigh rho = 0.541, *p* = 0.013). The strongest correlation was between calf muscle fat fraction and the combined lower limb motor symptoms and strength sub‐scores of the CMTES, (rho‐0.61, *p* < 0.001) as might be expected given their direct functional relationship (Fig. 2B). Thigh muscle fat fraction showed similar correlations with total knee MRC grade showing the strongest correlation. The patient subgroups showed overall similar correlations, with CMT2A showing stronger correlations at thigh level reflecting levels of fat accumulation at this level than the other patient subgroups. There was a highly significant moderate negative correlation between calf muscle fat fraction and sum ankle MRC grade in all three patient groups (Fig. 2C). The relationship between calf muscle fat fraction and NEFL levels was inconsistent: there was a positive correlation in controls (rho = 0.63, *p* = 0.02), a negative correlation in CMT1B patients (rho = −0.62, *p* = 0.003) and no correlation in CMTX1 and CMT2A groups (Table 4).

**Table 3:** Cross‐sectional MRI‐clinical correlations.

Clinical measure	CMTX1		CMT1B		CMT2A		All patients	
	Thigh FF	Calf FF	Thigh FF	Calf FF	Thigh FF	Calf FF	Thigh FF	Calf FF
Age	0.22; *p* = 0.329	**0.482; *p*** = **0.023**	0.28; *p* = 0.219	**0.651; *p*** = **0.003**	0.27; *p* = 0.254	**0.561; *p*** = **0.01**	0.160; *p* = 0.199	**0.362; *p*** = **0.004**
CMTESv2	0.09; *p* = 0.695	**0.425; *p*** = **0.049**	**0.535; *p*** = **0.012**	**0.719; *p*** = **0.001**	**0.459; *p*** = **0.042**	0.280; *p* = 0.234	**0.355; *p*** = **0.004**	**0.405; *p*** = **0.001**
LLM (CMTESv2)	0.25; *p* = 0.262	**0.560; *p*** = **0.007**	0.4; *p* = 0.069	**0.714; *p*** = **0.001**	**0.700; *p*** = **0.001**	**0.588; *p*** = **0.006**	**0.504; *p*** < **0.001**	**0.607; *p*** < **0.001**
CMTESv2‐R	0.06; *p* = 0.806	**0.42; *p*** = **0.050**	**0.529; *p*** = **0.014**	**0.729; *p*** < **0.001**	**0.544; *p*** = **0.013**	0.350; *p* = 0.127	**0.349; *p*** = **0.005**	**0.430; *p*** = **0.001**
LLM‐R (CMTESv2)	0.22; *p* = 0.33	**0.533; *p*** = **0.011**	0.38; *p* = 0.086	**0.664; *p*** = **0.002**	**0.700; *p*** = **0.001**	**0.588; *p*** = **0.006**	**0.456; *p*** < **0.001**	**0.574; *p*** < **0.001**
MRC (0–70)	−0.35; *p* = 0.112	**−0.655; *p*** = **0.001**	−0.28; *p* = 0.226	**−0.578; *p*** = **0.01**	**−0.683; *p*** = **0.001**	−0.330; *p* = 0.155	**−0.438; *p*** < **0.001**	**−0.481; <0.001**
MRCK (0–20)	NA	NA	0.19; *p* = 0.413	0.13; *p* = 0.582	**−0.773; *p*** < **0.001**	−0.400; *p* = 0.083	**−0.430; *p*** < **0.001**	−0.24; *p* = 0.057
MRCA (0–40)	**−0.530; *p*** = **0.011**	**−0.659; *p*** = **0.001**	−0.24; *p* = 0.298	**−0.561; *p*** = **0.012**	**−0.705; *p*** = **0.001**	**−0.444; *p*** = **0.050**	**−0.497; *p*** < **0.001**	**−0.561; *p*** < **0.001**
ONLS (0–12)	−0.06; *p* = 0.792	0.06; *p* = 0.777	**0.445; *p*** = **0.043**	0.41; *p* = 0.081	0.38; *p* = 0.112	0.19; *p* = 0.442	0.19; *p* = 0.141	0.15; *p* = 0.262
CMTHI (0–100)	0.06; *p* = 0.793	0.13; *p* = 0.579	0.43; *p* = 0.053	0.45; *p* = 0.053	0.02; *p* = 0.920	−0.03; *p* = 0.905	**0.326; *p*** = **0.010**	0.274; *p* = 0.034

Spearman rank correlation coefficients are given with *p*‐value, significant correlations in bold. NA: not applicable as all subjects had the same MRC Knee score of 20.

CMTESv2, Charcot‐Marie‐Tooth disease Examination score version 2; CMTESv2‐R, CMTESv2‐Rasch modified; CMTHI, CMT health index; LLM, lower limb motor component; MRC, Medical Research Council Sum Score; MRCA, MRC sum score of ankle; MRCK, MRC sum score of knee; ONLS, overall neuropathy limitation score.

**Table 4:** NEFL results.

	Controls			CMTX1			CMT1B			CMT2A		
NEFL (ng/L)	*n*	*Mean* ± *SD* (Range)	*p*	*n*	*Mean* ± *SD* (Range)	*p*	*n*	*Mean* ± *SD* (Range)	*p*	*n*	*Mean* ± *SD* (Range)	*p*
Baseline	14	6.6 ± 3.2 (3.5–15.9)	NA	21	13.6 ± 4.6 (5.9–20.5)	0.007	21	18.8 ± 8.8 (4.8–38.0)	<0.001	20	17.3 ± 5.4 (9.3–29.3)	<0.001
Annual change	14	0.3 ± 1.2 (−1.2–3.3)	0.34	17	−1.4 ± 2.4 (−6.7–2.0)	0.09	20	−1.4 ± 3.4 (−8.7–6.0)	0.07	18	−0.3 ± 2.8 (−6.9–4.0)	0.90

**Correlation of NEFL**	***n***	***R***	***p***	***n***	***R***	***p***	***n***	***R***	***p***	***n***	***R***	***p***
With age	14	0.76	0.002	21	−0.24	0.30	21	0.54	0.01	20	0.15	0.53
With calf muscle FF	14	0.63	0.02	21	−0.62	0.003	19	0.25	0.30	19	0.15	0.54

Baseline and annualised change values, and Spearman rank correlation with age and fat fraction are given in patient and control groups. *p*‐Value for baseline patient values are compared with control (Tukey HSD), while for change values are paired Student *t*‐tests.

There was a positive correlation between age and calf muscle fat fraction in all three patient groups with highest fat fraction seen in the CMT2A patient group. Control subjects' fat fraction remained low across all ages (Fig. 2D).

### Longitudinal data

Follow up assessments were undertaken in all but six subjects, most commonly due to subject choice. Overall between baseline and follow up there were three calf and two thigh fraction maps which could not be analysed due to movement artefact or other technical problems. Due to the COVID pandemic, the follow up interval was sometimes longer than the planned 365 days. The median interval was 396 days (IQR 371–504 days, range 350–978 days). All longitudinal data were divided by the interval in years to give annualised rates of change for analysis.

Annualised change in clinical measures are summarised in Table 5. There was a significant change seen in the CMTESv2 and CMTESv2‐R in the CMTX1 patient group only, however, this was not reflected in other measures or statistically significant in the other subgroups. No significant change of NEFL was seen in patient groups or controls (Table 4). Baseline NEFL did not correlate with subsequent change in fat fraction.

**Table 5:** Annualised change in clinical measures.

	CMTX1	CMT1B	CMT2A	All patients
CMTESv2	**0.71** ± **1.12** (***p*** = **0.005**)	0.4 ± 1.13 (*p* = 0.23)	0.05 ± 2.09 (*p* = 0.92)	**0.39** ± **1.51** (***p*** = **0.04**)
CMTESv2‐R	**0.85** ± **1.55** (***p*** = **0.012**)	0.69 ± 1.42 (*p* = 0.08)	−0.03 ± 3.12 (*p* = 0.95)	0.51 ± 2.16 (*p* = 0.06)
MRC	0.3 ± 1.3 (*p* = 0.67)	−0.22 ± 1.35 (*p* = 0.33)	0.78 ± 2.68 (*p* = 0.21)	0.28 ± 1.89 (*p* = 0.12)
CMT‐HI	−0.49 ± 8.61 (*p* = 0.507)	−2.34 ± 9.79 (*p* = 0.27)	0.08 ± 7.86 (*p* = 0.88)	−0.94 ± 8.71 (*p* = 0.29)
ONLS	**0.73** ± **1.31** (***p*** = **0.031**)	−0.1 ± 1.07 (*p* = 0.53)	0.35 ± 0.92 (*p* = 0.37)	**0.32** ± **1.15** (***p*** = **0.02**)

Shown as mean change ± *SD* change (*p*‐value) significant changes in bold.

Longitudinal MRI data are given in Table 2. There was no significant change of any of the MRI parameters in any of the matched control groups. All CMT types showed significant increases in calf muscle fat fraction over a standardised 12‐month interval: +2.0 ± 2.0%ff in CMTX1 and +1.6 ± 2.1%ff in both CMT1B and CMT2A. There was also significant progression of atrophy with between 3.1 and 4.6% reductions in calf muscle cross‐sectional area and between 6.6% and 7.6% decline in contractile cross‐sectional area. All of these parameters showed good responsiveness, with highest SRM for fat fraction: 1.02 for CMTX1, 0.78 for CMT1B and 0.76 for CMT2A.

At thigh level there was no significant change over 12 months in the CMTX1 group, while the CMT1B group showed a significant reduction in thigh contractile cross‐sectional area (−3.5 ± 6.6%, SRM = −0.54, *p* = 0.03). The CMT2A patients showed significant change over 12 months in all three MRI metrics at thigh level, with highest responsiveness in contractile cross‐sectional area (−5.2 ± 5.2%, SRM = −1.01, *p* < 0.001).

We then stratified patients by baseline fat fraction at each anatomical level based on our previous study in CMT1A^33^ into normal or near normal fat fraction (<10%), intermediate fat fraction (10–70%) and end‐stage fat fraction (>70%). These data are summarised in Table 6. Greatest change and highest responsiveness were seen in patients with intermediate fat fraction at baseline, for example those with between 10 and 70% fat fraction in calf muscles at baseline showed 2.7 ± 2.3%ff increase in fat fraction over 12 months (*p* < 0.001, SRM = 1.20) and 7.9 ± 7.7% reduction in contractile cross‐sectional area (*p* < 0.001, SRM = −1.04). Similarly, at thigh level those with intermediate fat fraction values showed significant progression of fat fraction (1.7 ± 2.1%ff, *p* = 0.01, SRM = 0.82) and reduction in contractile cross‐sectional area (−6.57 ± 5.36%, *p* = 0.01, SRM = −1.23).

**Table 6:** Annual change in MRI data in all patients grouped by baseline fat fraction.

		Change fat fraction (%ff)				Change CSA (% of baseline)				Change cCSA (% of baseline)			
Baseline Calf FF	*n*	*Mean*	*SD*	*p*	*SRM*	*Mean*	*SD*	*p*	*SRM*	*Mean*	*SD*	*p*	*SRM*
<10%	14	1.3	2.0	0.04	0.63	−3.6	6.7	0.05	−0.54	−4.6	7.1	0.03	−0.65
10%–70%	38	2.7	2.3	<0.001	1.20	−4.4	6.9	<0.001	−0.64	−7.9	7.7	<0.001	−1.04
>70%	6	2.1	4.5	0.31	0.46	−0.8	10.4	0.59	−0.07	−6.6	19.9	0.19	−0.33

Baseline Thigh FF	*n*	*Mean*	*SD*	*p*	*SRM*	*Mean*	*SD*	*p*	*SRM*	*Mean*	*SD*	*p*	*SRM*
<10%	44	0.1	0.4	0.15	0.32	−2.88	6.64	0.01	−0.43	−3.02	6.6	0.006	−0.46
>10%	14	1.7	2.1	0.01	0.82	−4.48	5.9	0.02	−0.76	−6.57	5.36	0.01	−1.23

Values are standardised to be change over 12 months. *p*‐Values are Student paired *t*‐tests for patient groups between baseline and follow up.

cCSA, contractile cross‐sectional area; CSA, total cross‐sectional area; FF, muscle fat fraction; SRM, standardised response mean.

There were no overall significant correlations between change in clinical measures and fat fraction, which is not unexpected as significant change in the clinical measures were not found.

## Discussion

Calf fat fraction measured using three‐point Dixon quantitative MRI has previously been shown to have large responsiveness over 12 months in patients with CMT1A and HSN1.^32^, ^33^, ^34^ We have expanded these findings to three other common CMT subtypes in a multi‐centre study across two different MRI vendors. Quantitative MRI of muscle fat fraction and contractile cross‐sectional area showed large responsiveness over 12 months in calf muscles in all three subgroups, while CMT2A patients also showed progression in thigh level muscles. MRI metrics have high validity with highly significant moderate correlation with clinical measures, but show superior responsiveness to these measures, making adequately powered clinical trials possible.

While broadly speaking the findings in these three CMT subtypes were similar, and were similar to the findings in CMT1A, there are some notable differences. The pattern of muscles involved within the calf was different in CMT2A with greater posterior compartment involvement than in CMT1B or CMTX1 in this study, and different to what we previously reported in CMT1A.^32^ However, this greater posterior compartment is noted clinically, and has previously been reported in qualitative muscle MRI by Chung and colleagues in CMT2A.^35^

When severely affected, all muscles show high levels of fat fraction in all CMT subtypes. Thus by utilising mean fat fraction across all calf muscles, a consistent increment in fat fraction is seen regardless of CMT subtype or severity, as long as the muscle tissue does not exhibit normal or end‐stage fat fraction. CMT2A patents had more severe motor involvement, especially proximal lower limb involvement than the other two subtypes, and consequently fat fraction was greater at both thigh and calf level. CMT2A patients also showed significant progression in fat fraction at thigh level, unlike CMTX1, CMT1B in this study or CMT1A previously. Muscle atrophy is also particularly marked in CMT2A with calf muscle cross‐sectional area just over half that of the control group (mean 61.0 vs. 119.3 cm^2^).

This study demonstrates that quantitative MRI of muscle fat fraction is a highly responsive outcome measure in three additional common inherited neuropathies: CMTX1, CMT1B and CMT2A. The magnitude of progression is similar to the progression seen previously in CMT1A^32^, ^33^ and HSN1.^34^

Indeed, the key factor which determines outcome measure responsiveness is disease severity rather than subtype. In this study progression is greatest for those with fat fraction between 10 and 70% at baseline as we have previously shown in CMT1A^33^ and HSN1.^34^ If we consider the more severely affected subgroup CMT2A, or all patients who were severely affected, thigh muscle fat fraction showed significant progression and was responsive as an outcome measure (SRM 0.67 CMT2A patients; SRM 0.82 all patients thigh FF > 10%). Furthermore, in this study, percentage change in contractile cross‐sectional area proved similarly or more responsive than muscle fat fraction, and could also be used as an outcome measure, especially if an intervention was expected to have an effect on muscle size as well as slowing progression of fat accumulation. Ultimately the best outcome measure for a clinical trial will depend on the exact patient group and intervention being studied; however, these data demonstrate that responsiveness can be increased by either selecting a more homogenous population, or using muscle severity specific outcome measures. This same approach has been applied to quantitative muscle MRI in fascioscapulohumeral dystrophy, where muscles with intermediate fat replacement are being used as an outcome measure in Phase 3 clinical trial of losmapimod.^38^

This study further validates quantitative MRI as an outcome measure in CMT by strong correlations with clinical measures including the CMTESv2, CMTESv2‐R and MRC scores in all patient groups. The strongest correlations were seen between MRI and directly linked lower limb motor functions such as the lower limb motor components of the CMTESv2 with calf fat fraction, and the total MRC score at the knee with thigh muscle fat fraction. NEFL results were significantly elevated in all patient groups compared with controls but did not change significantly over 12 months. However similar to previous longitudinal studies, the changes in clinical measures were not significant over this short length of time in this number of patients, and longitudinal correlations between clinical and MRI measures to confirm longitudinal validity would therefore require larger patient groups or longer duration of follow up.

This study benefits from a low rate of patient drop out and a well‐defined protocol meaning few scans were lost due to artefact. This was due to training and site qualification prior to commencing the study and ongoing quality control of scans throughout which will be vital if MRI is used as an outcome measure in a multisite clinical trial. However, due to the COVID pandemic halting observational studies, some patients had a longer follow up interval than 12 months, which we mitigated against by adjusting change values to an annualised rate. Other limitations in the study are that the number of patients in each subgroup, which limited more in‐depth analysis for example baseline stratification by age or disease severity in the subgroups. However the number of participants was more than adequate to show highly significant change over 12 months and allow power calculations for future clinical trials where quantitative muscle MRI is an outcome measure.

In summary, it is now established across all common subtypes of CMT, accounting for up to 90% of genetically confirmed cases, that quantitative MRI of lower limb muscle fat fraction represents an outcome measure with much greater sensitivity to detect change than current clinical outcome measures. All these forms of CMT, like most types of CMT are characterised by a length dependent motor and sensory neuropathy and it is likely that quantitative MRI lower limb muscle fat fraction will be a responsive outcome measure for all types of length dependent CMT with motor involvement. Furthermore, this study applies MRI quantification of lower limb muscle fat across two sites/countries/MRI vendors with centralised analysis demonstrating it is feasible in multi‐site clinical trials. While we recognise the need to correlate this MRI biomarker with clinical outcome measures over a longer period of follow up, this study suggests that the use of quantitative MRI lower limb muscle fat fraction will be a valuable outcome measure for upcoming clinical trials across many types of CMT.

## Author Contributions

Carolynne M. Doherty, Jasper M. Morrow, Riccardo Zuccarino, Paige Howard, Stephen Wastling, Tarek A. Yousry, Daniel Thedens, John Thornton, Michael E. Shy and Mary M. Reilly contributed to the conception and design of the study; Carolynne M. Doherty, Jasper M. Morrow, Riccardo Zuccarino, Paige Howard, Stephen Wastling, Menelaos Pipis, Nick Zafeiropoulos, Katherine J. Stephens, Tiffany Grider, Shawna M. E. Feely, Evelin Milev, Emma Nicolaisen, Magdalena Dudzeic, Amy McDowell, Nuran Dilek, Francesco Muntoni, Alexander M. Rossor, Sachit Shah, Matilde Laura, Tarek A. Yousry, Daniel Thedens, John Thornton, Michael E. Shy and Mary M. Reilly contributed to the acquisition and analysis of data; Carolynne M. Doherty, Jasper M. Morrow, Stephen Wastling, John Thornton, Michael E. Shy and Mary M. Reilly contributed to drafting the text or preparing the figures.

## Funding Information

This study was funded by the Muscular Dystrophy Association (MDA510281). M.M.R. is grateful to the Medical Research Council (MRC MR/S005021/1), the National Institutes of Neurological Diseases and Stroke and office of Rare Diseases (U54NS065712 and 1UOINS109403‐01 and R21TROO3034) and the Charcot Marie Tooth Association (CMTA) for their support.

## Conflict of Interest Statement

The authors report no competing interests.

## Data Availability

The data that support these study finding are available from the corresponding author upon reasonable request.

## References

[1] Lupski JR , de Oca‐Luna RM , Slaugenhaupt S , et al. DNA duplication associated with Charcot‐Marie‐tooth disease type 1A. Cell. 1991;66:219‐232. 1677316 10.1016/0092-8674(91)90613-4

[2] Raeymaekers P , Timmerman V , Nelis E , et al. Duplication in chromosome 17p11.2 in Charcot‐Marie‐tooth neuropathy type 1a (CMT 1a). Neuromuscul Disord. 1991;1(2):93‐97. 1822787 10.1016/0960-8966(91)90055-w

[3] Reilly MM , Murphy SM , Laurá M . Charcot‐Marie‐Tooth Disease. JPNS. 2011;16:1‐14. 10.1111/j.1529-8027.2011.00324.x 21504497

[4] Fridman V , Bundy B , Reilly MM , et al. CMT subtypes and disease burden in patients enrolled in the inherited neuropathies consortium natural history study: a cross‐sectional analysis. J Neurol Neurosurg Psychiatry. 2015;86(8):873‐878. 25430934 10.1136/jnnp-2014-308826 PMC4516002

[5] Panosyan FB , Laura M , Rossor AM , et al. Cross‐sectional analysis of a large cohort with X‐linked Charcot‐Marie‐tooth disease (CMTX1). Neurology. 2017;89(9):927‐935. 28768847 10.1212/WNL.0000000000004296 PMC5577965

[6] Siskind CE , Murphy SM , Ovens R , Polke J , Reilly MM , Shy ME . Phenotype expression in women with CMT1X. J Peripher Nerv Syst. 2011;16(2):102‐107. 21692908 10.1111/j.1529-8027.2011.00332.x

[7] Shy ME , Jáni A , Krajewski K , et al. Phenotypic clustering in MPZ mutations. Brain. 2004;127:371‐384. 14711881 10.1093/brain/awh048

[8] Sanmaneechai O , Feely S , Scherer SS , et al. Genotype‐phenotype characteristics and baseline natural history of heritable neuropathies caused by mutations in the MPZ gene. Brain. 2015;138(11):3180‐3192. 26310628 10.1093/brain/awv241 PMC4643641

[9] Chandhok G , Lazarou M , Neumann B . Structure, function, and regulation of mitofusin‐2 in health and disease. Biol Rev. 2018;93(2):933‐949. 29068134 10.1111/brv.12378 PMC6446723

[10] Chen H , Vermulst M , Wang YE , et al. Mitochondrial fusion is required for mtdna stability in skeletal muscle and tolerance of mtDNA mutations. Cell. 2010;141:280‐289. 20403324 10.1016/j.cell.2010.02.026 PMC2876819

[11] Braathen GJ . Genetic epidemiology of Charcot‐Marie‐tooth disease. Acta Neurol Scand [Internet]. 2012;126(S193):iv‐22. 10.1111/ane.12013 23106488

[12] Murphy SM , Laura M , Fawcett K , et al. Charcot–Marie–tooth disease: frequency of genetic subtypes and guidelines for genetic testing. J Neurol Neurosurg Psychiatry. 2012;83(7):706‐710. 22577229 10.1136/jnnp-2012-302451 PMC3736805

[13] Saporta ASD , Sottile SL , Miller LJ , Feely SME , Siskind CE , Shy ME . Charcot‐marie‐tooth disease subtypes and genetic testing strategies. Ann Neurol. 2011;69:22‐33. 21280073 10.1002/ana.22166 PMC3058597

[14] Boutary S , Echaniz‐Laguna A , Adams D , et al. Treating PMP22 gene duplication‐related Charcot‐Marie‐tooth disease: the past, the present and the future. Transl Res. 2021;227:100‐111. 32693030 10.1016/j.trsl.2020.07.006

[15] Pareyson D , Saveri P , Pisciotta C . New developments in Charcot‐Marie‐tooth neuropathy and related diseases. Curr Opin Neurol. 2017;30(5):471‐480. 28678038 10.1097/WCO.0000000000000474

[16] Rossor AM , Tomaselli P , Reilly MM . Recent advances in the genetic neuropathies. Curr Opin Neurol. 2016;4(1):139‐148. 10.1097/WCO.0000000000000373 PMC5130159 27584852

[17] Pisciotta C , Saveri P , Pareyson D . Updated review of therapeutic strategies for Charcot‐Marie‐tooth disease and related neuropathies. Expert Rev Neurothe. 2021;21(6):701‐713. 10.1080/14737175.2021.1935242 34033725

[18] Thenmozhi R , Lee JS , Park NY , Choi BO , Bin HY . Gene therapy options as new treatment for inherited peripheral neuropathy. Exp Neurobiol. 2020;29(3):177‐188. 32624504 10.5607/en20004 PMC7344374

[19] Husted JA , Cook RJ , Farewell VT , Gladman DD . Methods for assessing responsiveness: a critical review and recommendations. J Clin Epidemiol. 2000;53:459‐468. 10812317 10.1016/s0895-4356(99)00206-1

[20] Reilly MM , Herrmann DN , Pareyson D , et al. Trials for slowly progressive neurogenetic diseases need surrogate endpoints. Ann Neurol. 2023;93(5):906‐910. doi:10.1002/ana.26633 36891823 PMC10192108

[21] Liang MH , Fossel AH , Larson MG . Comparisons of five health status instruments for orthopedic evaluation. Med Care. 1990;28:632‐642. 2366602 10.1097/00005650-199007000-00008

[22] Shy ME , Blake J , Krajewski K , et al. Reliability and validity of the CMT neuropathy score as a measure of disability. Neurology. 2005;64(7):1209‐1214. 15824348 10.1212/01.WNL.0000156517.00615.A3

[23] Lewis RA , McDermott MP , Herrmann DN , et al. High‐dosage ascorbic acid treatment in charcot‐marie‐tooth disease type 1A results of a randomized, double‐masked, controlled trial. JAMA Neurol. 2013;70:981‐987. 23797954 10.1001/jamaneurol.2013.3178 PMC3752369

[24] Pareyson D , Reilly MM , Schenone A , et al. Ascorbic acid in charcot‐marie‐tooth disease type 1A (CMTTRIAAL and CMT‐TRAUK): a double‐blind randomised trial. Lancet Neurol. 2011;10:320‐328. 21393063 10.1016/S1474-4422(11)70025-4 PMC3154498

[25] Murphy SM , Herrmann DN , McDermott MP , et al. Reliability of the CMT neuropathy score (second version) in Charcot‐Marie‐tooth disease. J Peripher Nerv Syst. 2011;16:191‐198. 22003934 10.1111/j.1529-8027.2011.00350.x PMC3754828

[26] Sadjadi R , Reilly MM , Shy ME , et al. Psychometrics evaluation of Charcot‐Marie‐tooth neuropathy score (CMTNSv2) second version, using Rasch analysis. J Peripher Nerv Syst. 2014;19:192‐196. 25400013 10.1111/jns.12084 PMC4303498

[27] Burns J , Ouvrier R , Estilow T , et al. Validation of the CMT pediatric scale as an outcome measure of disability. Ann Neurol. 2012;71(5):642‐652. 22522479 10.1002/ana.23572 PMC3335189

[28] Mandarakas MR , Menezes MP , Rose KJ , et al. Development and validation of the Charcot‐Marie‐tooth disease infant scale. Brain. 2018;141(12):3319‐3330. 30476010 10.1093/brain/awy280 PMC6312041

[29] Fridman V , Sillau S , Acsadi G , et al. A longitudinal study of CMT1A using Rasch analysis based CMT neuropathy and examination scores. Neurology. 2020;94(9):e884. 32047073 10.1212/WNL.0000000000009035 PMC7238948

[30] Fridman V , Sillau S , Bockhorst J , et al. Disease progression in Charcot–Marie–tooth disease related to MPZ mutations: a longitudinal study. Ann Neurol. 2022;93:563‐576. 36203352 10.1002/ana.26518 PMC9977145

[31] Pipis M , Feely SME , Polke JM , et al. Natural history of Charcot‐Marie‐tooth disease type 2A: a large international multicentre study. Brain. 2020;143:3589‐3602. 33415332 10.1093/brain/awaa323 PMC7805791

[32] Morrow JM , Sinclair CDJ , Machado PM , et al. MRI biomarker assessment of neuromuscular disease progression: a prospective observational cohort study. Lancet Neurol [Internet]. 2016;15(15):65‐77. 26549782 10.1016/S1474-4422(15)00242-2 PMC4672173

[33] Morrow JM , Evans MRB , Grider T , et al. Validation of MRC Centre MRI calf muscle fat fraction protocol as an outcome measure in CMT1A. Neurology. 2018;91(12):e1125‐e1129. doi:10.1212/WNL.0000000000006214 30120135 PMC6161551

[34] Kugathasan U , Evans MRB , Morrow JM , et al. Development of MRC Centre MRI calf muscle fat fraction protocol as a sensitive outcome measure in hereditary sensory neuropathy type 1. J Neurol Neurosurg Psychiatry. 2019;90:895‐906. 30995999 10.1136/jnnp-2018-320198

[35] Chung KW , Suh BC , Shy ME , et al. Different clinical and magnetic resonance imaging features between Charcot‐Marie‐tooth disease type 1A and 2A. Neuromuscul Disord. 2008;18(8):610‐618. 18602827 10.1016/j.nmd.2008.05.012

[36] Glover GH , Schneider E . Three‐point Dixon technique for true water/fat decomposition with B0 inhomogeneity correction. Magn Reson Med. 1991;18:371‐383. 2046518 10.1002/mrm.1910180211

[37] Yushkevich PA , Piven J , Hazlett HC , et al. User‐guided 3D active contour segmentation of anatomical structures: significantly improved efficiency and reliability. Neuroimage. 2006;31(3):1116‐1128. 16545965 10.1016/j.neuroimage.2006.01.015

[38] Mellion ML , Widholm P , Karlsson M , et al. Quantitative muscle analysis in FSHD using whole‐body fat‐referenced MRI: composite scores for longitudinal and cross‐sectional analysis. Neurology. 2022;99(9):E877‐E889. 35750498 10.1212/WNL.0000000000200757

